# Effect of Serum Leptin on Weight Gain Induced by Olanzapine in Female Patients with Schizophrenia

**DOI:** 10.1371/journal.pone.0149518

**Published:** 2016-03-01

**Authors:** Nobuto Tsuneyama, Yutaro Suzuki, Kazushi Sawamura, Takuro Sugai, Naoki Fukui, Junzo Watanabe, Shin Ono, Mami Saito, Toshiyuki Someya

**Affiliations:** Department of Psychiatry, Niigata University Graduate School of Medical and Dental Sciences, Niigata, Japan; Medical University Innsbruck, AUSTRIA

## Abstract

**Background:**

Olanzapine (OLZ) treatment is associated with a high risk of weight gain, and may cause abnormalities in glycolipid metabolism. Therefore, the underlying mechanism of OLZ-related weight gain is needed to clarify but not yet been adequately determined. In recent years, adipocytokines such as leptin, adiponectin, and tumor necrosis factor (TNF)-α, which play important roles in energy homeostasis, have been suggested as biomarkers of weight gain. Here, we determined if baseline plasma concentrations of leptin, adiponectin, and TNF-α predict weight gain following OLZ treatment.

**Methods:**

We recruited 31 schizophrenia outpatients (12 men and 19 women, 28.8 ± 10.2 years old) that were unmedicated or on another antipsychotic monotherapy medication. Baseline body mass index (BMI) and plasma levels of leptin, adiponectin, and TNF-α were obtained. All patients started or were switched to OLZ monotherapy for a maximum of 1 year. BMI was also obtained at the endpoint.

**Results:**

Mean BMI change following OLZ treatment was 2.1 ± 2.7 kg/m^2^. BMI change from baseline to endpoint negatively-correlated with baseline leptin levels in female patients (r = −0.514, *P* = 0.024), but not male patients. Baseline adiponectin or TNF-α levels were not correlated with BMI change.

**Conclusion:**

Baseline plasma leptin can have an effect on subsequent weight gain following OLZ treatment in female patients with schizophrenia.

## Introduction

Pharmacological treatment options for schizophrenic disorder have increased considerably with the arrival of second-generation antipsychotics (SGAs). Among SGAs, olanzapine (OLZ) is effective and well-tolerated, and therefore frequently administered [1], although it is also most frequently associated with the side-effect of weight gain [2–3]. Weight gain can lead to glycolipid metabolic disorders and decreased treatment adherence [4], therefore predicting and preventing weight gain is desirable. However, individual variations in weight gain due to OLZ are large [5]. The mechanisms of variations are partly explained by the activity at histamine H_1_, serotonin 5-HT_2C_, 5-HT_2A_, muscarinic M_3_, adrenergic receptors [6], orexigenic/anorexigenic neuropeptides and their receptors [7], but have not yet been adequately explained, prediction and prevention are difficult.

In recent years, adipocytokines secreted by adipose cells, namely, leptin, adiponectin, and tumor necrosis factor (TNF)-α, have gained attention in the medical field, and are known to be involved in weight change through roles in food intake, energy consumption, and glycolipid metabolism [8]. Leptin increases in proportion to the amount of fat [9]. States of energy excess are associated with hyperleptinemia, but the hypothalamus is resistant or tolerant to the effects of increased leptin (dashed line). Energy deficiency results in hypoleptinemia. As a result, a complex neural circuit comprising orexigenic and anorexigenic signals is activated to increase food intake. Furthermore, for the same body fat, women have greater leptin concentrations than men, as a result of differences in sex hormones. Adiponectin, an antiatherogenic and antiinflammatory protein, enhances insulin sensitivity and is inversely correlated with obesity and visceral fat [10]. In addition, adiponectin leads to central nervous system-induced weight loss [11]. TNF-α, a cytokine widely known as a biological defense mechanism that acts via inflammation, increases lipolysis, inhibits adipogenesis, and decreases weight due to infection. TNF-α is significantly observed in adipose cells of obese individuals, and triggers insulin resistance [12]. Additionally, there are reports suggesting correlation between activation of the TNF-α system by OLZ and weight gain [13].

Based on these reports, we previously focused on the relationship between metabolic disorders due to SGA, and adipocytokines. By comparing a patient group using SGAs (including OLZ) with a healthy control group, we found lower adiponectin in blood serum in the SGA user group, and in contrast, higher leptin that correlated with weight [14]. Furthermore, switching from OLZ to aripiprazole treatment led to increased adiponectin and decreased TNF-α in serum [15], suggesting that serum adipocytokine levels may be related to OLZ-induced weight gain. Moreover, although the study sample size was small, baseline leptin concentration was shown to affect change in body mass index (BMI) after OLZ treatment [16]. Here, we examine the hypothesis that baseline serum concentrations of leptin, adiponectin, and TNF-α are related to post-OLZ use weight change.

## Methods

### Subjects

In total, 31 outpatients aged 18–60 years and diagnosed with schizophrenia based on the Diagnostic and Statistical Manual of Mental Disorders-IV-Text Revision (DSM-IV-TR) at the Department of Psychiatry of Niigata University Medical and Dental Hospital were recruited. Participants were required to have been untreated with antipsychotics over 4 weeks or receiving the same daily dose of single SGA treatment of aripiprazole, risperidone, blonanserin, or perospirone for at least 4 weeks. Participants were excluded if they met the following criteria: diabetes, dyslipidemia, endocrine disease, concurrent treatment with other antipsychotic agents, or concurrent treatment with any drugs other than benzodiazepines. The study was performed with approval from the Gene Ethics Committee of Niigata University Graduate School of Medical and Dental Sciences. All patients and/or their families were given thorough explanations prior to obtaining written consent.

### Methods for Drug Administration and Evaluation

At baseline, scores on the Brief Psychiatric Rating Scale (BPRS) were evaluated, and fasting blood samples were drawn after an overnight fast of at least 8 h to examine leptin, adiponectin, TNF-α and fasting blood glucose. Plasma samples were centrifuged at 4°C and stored at −80°C. Serum analyses were performed by standard methods (SRL, Inc., Japan). Participants’ BMI (weight in kilograms divided by the square of their height in meters) was calculated. Following baseline assessments, for individuals who were receiving antipsychotic medications without OLZ, the dosage was reduced to a 400-mg/day chlorpromazine equivalent over 2 weeks and then discontinued within 4weeks. In parallel, OLZ treatment was started at an initial dose of 5 mg/day and OLZ dose was adjusted every 2 to 4 weeks in the range of 20mg/day on the basis of clinical judgments. BMI and BPRS were calculated at the endpoint, which was defined as either the point after 1 year from baseline or at OLZ discontinuation for any reason.

### Statistical Analysis

BMI change was defined as the amount of subsequent BMI change from baseline to endpoint. The Shapiro–Wilk test was used to assess whether the data were normally distributed. Because almost all the data without smoker’s number were normally distributed, a Pearson-correlation analysis was performed between BMI change and following variables; age, baseline BMI, duration of illness, olanzapine-treatment period, daily olanzapine dose, fasting blood glucose at baseline, baseline BPRS, and baseline concentration of leptin, adiponectin, TNF-α. Spearman’s rank correlation test was performed between BMI change and smoker’s number. Using the variables significantly correlated with BMI change, a stepwise multiple regression analysis was performed to evaluate the effects of independent variables. Statistical significance threshold was defined as *P* < 0.05. SPSS-21.0 (IBM Japan, Ltd., Tokyo, Japan) was used for statistical calculations.

## Results

**Fig 1:**
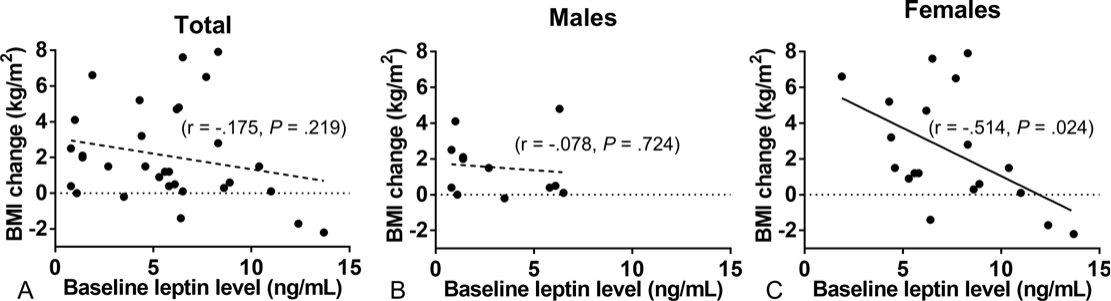
Relationship between BMI change following olanzapine treatment and baseline plasma leptin concentration. (A) In all patients, BMI change did not correlate with baseline leptin levels. (B) In male patients, BMI change did not correlate with baseline leptin levels. (C) In female patients, BMI change negatively-correlated with baseline leptin levels (*r* = -0.514, *P* = 0.024).

Clinical characteristics and baseline data of patients are shown in Table 1. At baseline, leptin and adiponectin levels in females were higher than in males, while TNF-α levels were higher in males than females. There was no significant difference between males and females in age, baseline BMI, duration of illness, treatment period of olanzapine, daily olanzapine dose, fasting blood glucose at baseline, baseline BPRS, and number of smokers. Nineteen patients were successfully treated with OLZ over 1 year. Twelve patients discontinued OLZ before 1 year because of insufficient or adverse effect. In either case, BMI and BPRS were calculated at the endpoint in all 31 patients. Changes in BMI and BPRS from baseline to endpoint are shown in Table 2. Mean BMI change following OLZ treatment was 2.1 ± 2.7 kg/m^2^, and did not correlate with any variables in any patients. However, in female patients, BMI change was negatively-correlated with baseline leptin levels (r = −.514, *P* = .024) (Fig 1) and olanzapine-treatment period (r = −.520, *P* = .027), but not correlated with other variables. Moreover, baseline leptin levels were not correlated with olanzapine-treatment period in in female patients. In male patients, BMI change was not correlated with any variables. We conducted a stepwise multiple regression analysis in female patients to evaluate the effects of baseline leptin levels and olanzapine-treatment period on BMI change. Higher baseline leptin level was found to contribute to lower BMI gain (adjusted R^2^ = .275, β = -.584, *P* = .046).

**Table 1:** Clinical characteristics and baseline data.

	Total (n = 31)	Male (n = 12)	Female (n = 19)	*P*
Drug, n				
None	19	9	10	-
Risperidone	8	3	5	-
Aripiprazole	2	0	2	-
Blonanserin	1	0	1	-
Perospirone	1	0	1	-
Duration of illness, year*	5.6 ± 5.9	6.4 ± 6.7	5.1 ± 5.5	NS^†^
Age, year*	28.8 ± 10.2	25.9 ± 8.7	30.7 ± 10.8	NS^†^
Smoker, n (%)	9 (29.0)	4 (33.3)	5 (26.3)	NS^‡^
BPRS*	32.6 ± 7.9	35.9 ± 6.2	30.0 ± 8.3	NS^†^
BMI, kg/m^2^*	20.7 ± 3.0	21.0 ± 3.6	20.5 ± 2.6	NS^†^
Leptin, ng/mL*	5.7 ± 3.5	3.1 ± 2.4	7.4 ± 3.0	< 0.001^†^
Adiponectin, μg/mL*	10.4 ± 4.8	7.5 ± 2.7	12.2 ± 5.0	0.02^†^
TNF-α, pg/mL*	1.7 ± 0.5	2.0 ± 0.7	1.5 ± 0.2	0.049^†^

*Data are expressed as mean ± standard deviation.

^†^Unpaired *t*-test was used to compare between males and females.

^‡^Chi-square test was used to compare between males and females.

Abbreviations: BPRS, Brief Psychiatric Rating Scale; BMI, body mass index; NS, not significant; TNF-α, tumor necrosis factor-α.

**Table 2:** BMI change following olanzapine treatment.

	Total (n = 31)			Male (n = 12)			Female (n = 19)		
	Baseline	Endpoint	*P*	Baseline	Endpoint	*P*	Baseline	Endpoint	*P*
Olanzapine dose, mg/day*	0.0 ± 0.0	12.4 ± 7.1	-	0.0 ± 0.0	13.8 ± 8.6	-	0.0 ± 0.0	11.6 ± 6.0	-
Duration of olanzapine medication, weeks*	0.0 ± 0.0	37.0 ± 27.5	-	0.0 ± 0.0	33.6 ± 33.5	-	0.0 ± 0.0	39.0 ± 24.0	-
BPRS*	32.6 ± 7.9	26.2 ± 7.3	.004^†^	35.9 ± 6.2	31.7 ± 6.2	NS^†^	30.0 ± 8.3	22.8 ± 5.7	.031^†^
BMI, kg/m^2^*	20.7 ± 3.0	22.8 ± 3.9	< .001^†^	21.0 ± 3.6	22.5 ± 3.9	.008^†^	20.5 ± 2.6	23.0 ± 3.9	.003^†^

*Data are expressed as mean ± standard deviation.

^†^Paired *t*-test was used.

Abbreviations: BPRS, Brief Psychiatric Rating Scale; BMI, body mass index; NS, not significant.

## Discussion

Our results show that body weight change in females may be associated with peripheral blood leptin levels prior to commencing OLZ treatment.

Wang *et al*. [16] used OLZ treatment for 14 days in nine (5 male and 4 female) patients with schizophrenia, and measured BMI along with blood leptin concentration prior to and following treatment. They observed a negative correlation between BMI change and leptin concentration prior to treatment, which corresponds with our finding in females. The mechanism by which body weight change is associated with blood leptin levels prior to OLZ treatment is not clear. Leptin acts on leptin receptors, causing strong suppression of food intake, and leading to accelerated energy consumption and maintained energy homeostasis [17]. It has been reported that OLZ administration results in decreased leptin receptors and increased leptin concentration [18]. It is possible that patients with low leptin concentrations may have higher sensitivity towards leptin receptors than patients with higher leptin concentrations. Therefore body weight maintenance and energy homeostasis may be more susceptible to the effect of declined leptin receptors due to OLZ administration, consequently leading to significant weight gain. In contrast, Monteleone *et al*. [19] treated 22 (13 male and 9 female) schizophrenic patients with clozapine, measuring BMI and blood leptin levels every 2 weeks for a maximum of 32 weeks, but found no relationship between leptin levels prior to treatment and body weight change. Our results do not correspond with their results; however, Monteleone *et al*. did not examine males and females separately, so the details are unknown.

We found that BMI change from an average OLZ administration period of 37 weeks was 2.1 kg/m^2^. The OLZ administration period varies greatly in past reports (6 weeks to approximately 2 years), with BMI increment due to OLZ treatment ranging from 0.91 to 4.9 kg/m^2^ [20–23], consistent with our results. Taking gender differences into consideration, we found that BMI change was greater in females: 1.5 kg/m^2^ in males and 2.5 kg/m^2^ in females (Table 2). Although it is known that OLZ causes weight gain in both males and females, BMI increment is greater in females compared with males [21, 23–24]. In rats, significant weight gain is only observed in female rats administered OLZ compared with controls [25–27]. An explanation for this has been proposed based on observed gender differences in the anorexigenic effect of leptin [28] and the number of leptin receptors expressed in the brain [29], which may affect the gender difference in body weight change. Furthermore, a gender difference in energy consumption [30–31] may also be involved. However, some clinical studies have observed no gender difference in BMI change [20, 32–33], and consistent conclusions in patients have not yet been shown.

We observed no relationship between weight gain and adiponectin or TNF-α levels prior to administration. Adiponectin increases insulin sensitivity and shows an inverse correlation with body weight and visceral fat [10]. TNF-α is an inflammatory cytokine that is known to cause insulin resistance [12]. To date, there have not been any reports indicating a relationship between adiponectin or TNF-α levels and subsequent body weight change, necessitating further studies in the future.

There are several limitations to our study. First, the sample size is small, therefore the result in male patients should be reanalyzed using larger samples. Second, medications prior to OLZ administration were not controlled, and an effect of the previous drug on weight may have remained even after commencing OLZ treatment. Third, the treatment period from commencing OLZ treatment to the endpoint varied. Further studies (e.g. on animals) are needed to reveal molecular mechanism of leptin-related olanzapine-induced weight gain.

## Conclusions

In this study, it was suggested that the serum leptin may have effect on weight gain in female patients following treatment with olanzapine. If leptin contribute to clarify the mechanism of OLZ-related weight gain, weight gain may be predicted prior to treatment, additional interventions such as nutrition education may be used from an early period of treatment, thereby preventing weight gain.
